# Cystic Duct Stump Calculus Mimicking Functional Dyspepsia: A Rare Cause of Post-cholecystectomy Syndrome

**DOI:** 10.7759/cureus.88833

**Published:** 2025-07-26

**Authors:** Binyam M Habte, Yoseph M Habte, Esimael M Abdu, Makida M Habte

**Affiliations:** 1 Department of Medicine, University of Gondar, Gondar, ETH; 2 Department of Medicine, Ethio Tebib Hospital, Addis Ababa, ETH; 3 Department of Surgery, Teklehaimanot General Hospital, Addis Ababa, ETH; 4 Department of Medicine, Bethel Medical College, Addis Ababa, ETH

**Keywords:** case report, completion cholecystectomy, cystic duct stump stone, functional dyspepsia mimic, post-cholecystectomy syndrome

## Abstract

Persistent gastrointestinal symptoms after cholecystectomy are not uncommon and are frequently attributed to functional disorders such as dyspepsia or gastritis. However, rare structural causes (such as retained calculi within the cystic duct stump or a remnant gallbladder) can mimic benign gastrointestinal conditions, particularly in the absence of classical biliary signs. We present the case of a 47-year-old female with a one-year history of postprandial epigastric discomfort, bloating, and dyspepsia. She had undergone an open cholecystectomy five years earlier for gallstone disease. Despite multiple courses of proton pump inhibitors and a normal upper gastrointestinal endoscopy, her symptoms persisted. Laboratory studies revealed mildly elevated alanine transaminase (ALT) (64 U/L), while other parameters, including complete blood count and *Helicobacter pylori *stool antigen, were normal. Abdominal ultrasound identified a 1.2 cm calculus within the cystic duct stump. Given the persistent symptoms and imaging findings, the patient underwent elective exploratory laparotomy, which confirmed the presence of dense adhesions and a retained stone. Completion cholecystectomy using a fundus-first approach was performed successfully, and the patient experienced complete symptom resolution postoperatively. This case highlights the importance of maintaining a high index of suspicion for retained biliary calculi in patients with persistent upper abdominal symptoms following cholecystectomy, especially when standard investigations such as endoscopy fail to identify a cause. A systematic diagnostic approach that includes targeted biliary imaging is essential to avoid misdiagnosis, reduce unnecessary treatments, and ensure timely surgical intervention when indicated.

## Introduction

Cholecystectomy, both open and laparoscopic, remains the standard treatment for symptomatic cholelithiasis and gallbladder-related disorders. Although generally safe and effective, a subset of patients continue to experience persistent or recurrent upper abdominal symptoms after surgery, a condition commonly referred to as post-cholecystectomy syndrome (PCS). PCS is reported to affect 10-30% of cholecystectomy patients and encompasses a broad range of gastrointestinal complaints, including epigastric pain, bloating, nausea, dyspepsia, and diarrhea [1,2].

Retained stones in the cystic duct remnant represent a rare but clinically significant cause of post-cholecystectomy syndrome. These may develop in a long residual cystic duct or gallbladder stump left behind inadvertently during difficult dissections. Patients can remain asymptomatic for years before presenting with nonspecific gastrointestinal complaints, often mimicking functional dyspepsia, which may delay diagnosis and appropriate management. Although data on the natural course of biliary stones is limited, studies estimate that retained or recurrent bile duct stones occur in approximately 1.2% to 14% of cases, with only about 0.3% becoming symptomatic. This low rate of clinical presentation contributes to cystic duct stump stones being frequently overlooked in the initial evaluation of persistent post-cholecystectomy symptoms [2].

We present a case of a 47-year-old female who was misdiagnosed with dyspepsia for over a year after cholecystectomy, but was ultimately found to have a 1.2-cm cystic duct stump stone. The case underscores the diagnostic challenge posed by retained biliary calculi in the post-cholecystectomy setting and highlights the importance of imaging and clinical suspicion in reaching the correct diagnosis.

## Case presentation

A 47-year-old female presented to the outpatient surgical clinic with a one-year history of persistent bloating and epigastric pain. The pain was described as dull, non-radiating, intermittent, and not related to meals. She denied nausea, vomiting, weight loss, hematemesis, or melena. There was no history of jaundice, fever, or changes in bowel habits. The symptoms had progressively affected her quality of life despite multiple courses of proton pump inhibitors (PPIs), which failed to provide any relief.

Her past medical history was notable for an open cholecystectomy performed five years prior at another institution due to symptomatic gallstone disease. She had no known history of hypertension, diabetes mellitus, tuberculosis, or other chronic illnesses. She reported no history of alcohol consumption, smoking, or recent medication changes.

On physical examination, the patient appeared well and in no acute distress. Vital signs were within normal limits: temperature 36.8°C, heart rate 78 bpm, respiratory rate 16 breaths/min, blood pressure 120/75 mmHg, and oxygen saturation 98% on room air. Abdominal examination revealed mild epigastric tenderness without guarding or rebound tenderness. Murphy’s sign was negative. No hepatosplenomegaly or palpable mass was appreciated.

Initial laboratory investigations were unremarkable, including complete blood count (CBC), renal function tests, and serum electrolytes. Liver function tests were largely within normal range, with the exception of a mildly elevated alanine transaminase (ALT) at 64 IU/L (normal <40 IU/L). Fasting blood sugar (FBS) was 113 mg/dl, consistent with impaired fasting glucose, and hemoglobin A1C (HbA1c) was 6%. Stool examination was negative for *Helicobacter pylori *antigen, ova, and parasites. Urinalysis was normal (Table 1).

**Table 1:** Laboratory results at presentation Impaired fasting glucose and elevated alanine transaminase (ALT) were the only abnormal findings; all other parameters were within normal limits.

	Result	Reference value
White Blood Cells	8,200/mm^3^	4,100-11,000/mm^3^
Neutrophil	65.6%	50-70%
Lymphocyte	28.2%	20-40%
Eosinophil	2%	0-6%
Hemoglobin	13.2 mg/dl	11-16 mg/dl
Platelet	405,000/mm^3^	100,000-430,000/mm^3^
Blood Urea Nitrogen	14 mg/dl	6-20 mg/dl
Creatinine	0.9 mg/dl	0.7-1.2 mg/dl
Aspartate Transaminase	35 mg/dl	0-37 mg/dl
Alanine Transaminase	64 mg/dl	0-42 mg/dl
Hepatitis B surface antigen	Negative	
Hepatitis C antibody	Negative	
Fasting blood sugar	113 mg/dl	74-106 mg/dl
Thyroid-stimulating hormone	0.6 µIU/ml	0.3-4.3µIU/ml
Hemoglobin A1C	6%	< 5.7%
Na+	138 mmol/L	135-145 mmol/L
K+	4.2 mmol/L	3.5-5.0 mmol/L
Cl-	102 mmol/L	98-106 mmol/L
Ca^2+^(Total)	2.4 mmol/L	2.1-2.6 mmol/L

To investigate persistent upper gastrointestinal (GI) symptoms, an upper GI endoscopy was performed, revealing normal mucosa throughout the esophagus, stomach, and duodenum. No ulcers, erosions, or signs of gastritis were noted. Given the persistence of symptoms, an abdominal ultrasound was performed, which showed the gallbladder had been previously removed. However, a 1.2 cm echogenic focus with posterior acoustic shadowing was seen in the cystic duct stump, suggesting a retained calculus. The common bile duct (CBD) was not dilated, and no intrahepatic biliary dilation was observed.

In light of the findings and the chronicity of her symptoms, a decision was made to proceed with elective surgical exploration for suspected retained cystic duct calculus.

**Figure 1:**
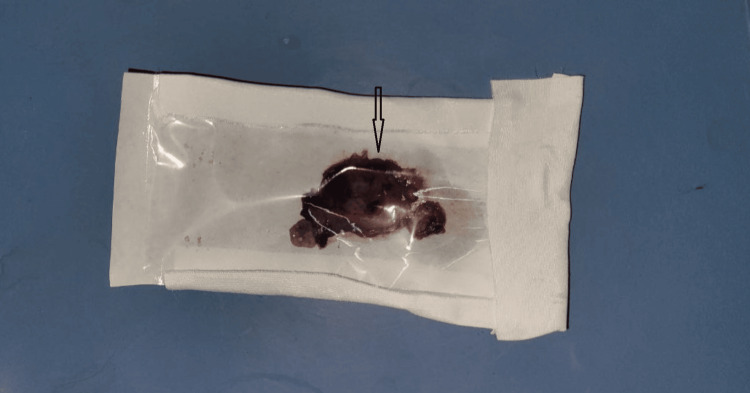
Shrunken gall bladder remnant and excised cystic duct stump containing retained stone The excised cystic duct stump contained a retained calculus. The specimen was obtained during the completion cholecystectomy performed to resolve persistent dyspeptic symptoms following the previous cholecystectomy.

Under general anesthesia, a Kocher’s incision (right subcostal incision) was used to access the peritoneal cavity. Intraoperatively, dense adhesions involving the omentum, hepatic flexure of the colon, and the duodenum to the gallbladder fossa were encountered. Careful adhesiolysis was performed. A shrunken gallbladder remnant and cystic duct stump were identified. A fundus-first completion cholecystectomy was carried out, with ligation and division of the cystic duct and artery. Although operative field images were not captured or archived during the initial surgery, a photograph of the excised specimen was taken and has been included in this report for documentation purposes (Figure 1).

The patient tolerated the procedure well, and there were no intraoperative or immediate postoperative complications. She was discharged on postoperative day two in stable condition with adequate pain control. At a follow-up visit two weeks later, the patient reported complete resolution of her epigastric discomfort and bloating. A glycemic control plan was initiated for her newly diagnosed diabetes mellitus. 

## Discussion

The diagnostic challenges of upper abdominal symptoms in post-cholecystectomy patients lie in the broad differential diagnosis and frequent overlap between functional and organic causes. While cholecystectomy is intended to resolve symptoms associated with gallstone disease, not all patients experience complete relief. When symptoms such as dyspepsia, bloating, or upper abdominal discomfort persist or recur, clinicians often attribute them to functional gastrointestinal disorders, particularly in the absence of jaundice, abnormal liver function tests, or overt biliary colic. However, such assumptions can lead to misdiagnosis and delayed recognition of less common biliary complications, such as retained stones in the cystic duct remnant or gallbladder remnant [3,4].

In the case presented, the patient reported ongoing dyspeptic symptoms for one year after undergoing an open cholecystectomy five years prior to presentation. These symptoms were initially interpreted as functional in nature due to their nonspecific character and the absence of abnormalities on upper gastrointestinal endoscopy. Moreover, the lack of classic signs of biliary obstruction, such as jaundice, biliary colic, or signs of cholangitis, further diverted clinical suspicion away from a biliary source. Despite being managed empirically with proton pump inhibitors (PPIs), her symptoms persisted, prompting further investigation. Abdominal ultrasound ultimately revealed a 1.2 cm calculus located within the residual cystic duct. This finding highlights how retained biliary stones can mimic functional gastrointestinal disorders, often delaying accurate diagnosis and leading to ineffective treatment and patient frustration.

The occurrence of retained or recurrent stones within the cystic duct remnant, while uncommon, is a recognized postoperative complication. Such stones may be the result of incomplete gallbladder excision, the presence of an elongated cystic duct remnant, or technical challenges encountered during surgery, particularly in the setting of inflammation or dense adhesions [5].

From a clinical perspective, patients with retained cystic duct stones may present with intermittent right upper quadrant discomfort, nausea, bloating, or dyspeptic symptoms, which may resemble more benign or functional gastrointestinal disorders such as gastritis. Unlike stones within the common bile duct, cystic duct remnant stones do not always lead to jaundice or significant alterations in liver enzyme levels [6]. In the present case, the patient’s mildly elevated ALT level was non-specific and, in isolation, unlikely to prompt further biliary investigation without supporting clinical or imaging findings. However, in the context of post-cholecystectomy symptoms, this biochemical abnormality may reflect early or low-grade biliary irritation due to a retained cystic duct stump stone. Other potential causes of ALT elevation, such as viral hepatitis, medication-induced liver injury, and alcohol-related liver disease, were considered less likely given the patient’s unremarkable clinical history, lack of risk factors, and normal serologic workup.

Imaging modalities play a pivotal role in diagnosing this condition. While transabdominal ultrasonography remains the first-line diagnostic tool due to its accessibility and non-invasive nature, its accuracy can be influenced by the operator’s skill and patient factors. In more ambiguous or anatomically complex cases, magnetic resonance cholangiopancreatography (MRCP) or CT cholangiography can provide superior visualization of the biliary tree and remnant structures [7]. In our patient's case, the abdominal ultrasound clearly visualized the retained stone, and surgical exploration validated the imaging findings.

Definitive treatment of symptomatic retained cystic duct stones requires surgical removal. Whenever feasible, laparoscopic re-intervention is preferred due to its minimally invasive nature and shorter recovery time. However, in patients with prior open surgery or extensive intra-abdominal adhesions, a repeat open procedure may be necessary [8]. In this case, due to the presence of dense adhesions involving the omentum, duodenum, and colon, a laparotomy was performed. A fundus-first (top-down) approach facilitated dissection and safe identification of the cystic duct, allowing successful resection and symptom resolution.

This case reinforces the importance of maintaining a broad differential diagnosis in patients presenting with chronic upper abdominal symptoms following cholecystectomy. In particular, biliary causes should not be excluded solely based on non-classical presentations. A methodical approach, starting with history and physical examination, progressing through basic laboratory tests and targeted imaging, can ensure timely identification of structural causes and appropriate intervention [7]. Furthermore, the case highlights the importance of complete gallbladder removal during the initial surgery and meticulous ligation of the cystic duct to prevent the formation of long cystic duct remnants or gallbladder remnants, which are potential sources of future pathology [9].

Finally, this case also demonstrates the need to reassess diagnoses in patients unresponsive to empirical treatments. Assigning a functional diagnosis prematurely, without ruling out correctable structural conditions, can result in prolonged patient suffering, unnecessary medication use, and healthcare inefficiency [10]. In our patient's situation, accurate imaging ultimately revealed the correct etiology after a year of misdiagnosis, and definitive surgical management led to a successful outcome.

## Conclusions

This case reinforces the need for vigilance in evaluating persistent gastrointestinal symptoms after cholecystectomy. Retained biliary calculi, though rare, should remain on the differentials when symptoms are unexplained by routine investigations. Recognizing subtle presentations and pursuing appropriate imaging can lead to timely, curative intervention. This report adds to the limited literature on post-cholecystectomy calculi and emphasizes the clinical value of reconsidering prior surgical anatomy in patients with unresolved symptoms.
